# Standardized "Malhotra-Wig Vignettes" for Research in India : A Review with Full Text

**Published:** 2004

**Authors:** H K Malhotra, N N Wig

**Affiliations:** 1 Diplomat American Board of Psychiatry and Neurology is a Clinical Assistant Professor of Psychiatry in New Jersey Medical School, University of Medicine and Dentistry of New Jersey at Newark, USA; 2 Ex professor and chief of the department of Psychiatry of Postgraduate institute of medical Education and Research at Chandigarh, India


ORIGINAL ARTICLE *Indian Journal of Psychiatry, 2004, 46(I)52-64*

### Standardized "Malhotra-Wig Vignettes" for Research in India: A Review with Full Text

**H. K. Malhotra*[1], N.N. Wig[2]**

### Introduction

Vignettes (Synonyms: paper cases, hypothetical case histories, fictionalized narrative case summaries, psychopathology vignettes, standardized case histories) are simulations of real events which can be used in research studies to elicit subject's knowledge, attitudes or opinions according to how they state they would behave in the hypothetical situation depicted. Advantages associated with the use of vignettes as research tools include: the ability to collect information simultaneously from large numbers of subjects, to manipulate a number of variables at once in a manner that would not be possible in observation studies, absence of observer effect and avoidance of the ethical dilemmas commonly encountered during observation. Difficulties include problems establishing reliability and validity, especially external validity (Gould, 1996). Vignettes can be very useful research tools yielding valuable data when studying people's attitudes, perceptions and beliefs in social and nursing research (Hughes and Huby, 2002).

Peabody et al (2000) conducted a prospective trial comparing 3 methods for measuring the quality of care for 4 common outpatient conditions: (1) structured reports by standardized patients (SPs), trained actors who presented unannounced to physicians' clinics (the gold standard); (2) abstraction of medical records for those same visits; and (3) physicians' responses to clinical vignettes that exactly corresponded to the SPs' presentations. Their data indicated that quality of health care can be measured in an outpatient setting by using clinical vignettes. Vignettes appear to be a valid and comprehensive method that directly focuses on the process of care provided in actual clinical practice. Vignettes show promise as an inexpensive case-mix adjusted method for measuring the quality of care.

### Review of International Literature

The International literature reveals that multiple areas of investigations have used vignettes as a research tool. Galante et al (2003) published his review with a view to verifying, quantifying and analyzing the use of vignettes as a strategy for data collection. He researched the MEDLINE and LILACS systems, in the period from 1966 to 2000 and found five hundred eighty-two research works.

### Study of diagnostic practices of professionals using vignettes

Hjortso et al (1989) studied inter-rater reliability of psychiatric target syndromes and diagnoses and compared ICD-8, ICD-10 and DSM-III. Kitamura et al (1989) studied the reliability of conventional diagnosis and discrepancies in Research Diagnostic Criteria diagnosis in Japan. Hasui et al (1999) examined the reliability of the diagnoses of DSM-IV childhood mental disorders by Japanese psychologists, using 20 case vignettes. Stelmachers and Sherman (1990) used case vignette to rate reliability of global assessments of suicidality by the crisis workers. The reliability of the Global Assessment of Functioning scale (GAF) was studied by Bates et al (2002) and Oliver et al (2003) by using vignettes and asking the subjects to rate GAF.

Kee (2003) studied models of clinical judgment in novice and expert physicians. Fifty practitioners appraised 60 vignettes describing a child with an exacerbation of asthma and rated their propensities to admit the child.

Flanagan and Blashfield (2003) reported on gender bias on the diagnosis of personality disorders. They asked ratings of case vignettes with the sex of the patient being male or female. Hsieh and Kirk (2003) studied the effect of social context on psychiatrists' judgments of adolescent antisocial behavior. A representative sample of 483 psychiatrists in the United States read one of three experimentally manipulated vignettes depicting adolescent antisocial behavior and responded to questions concerning its nature, prognosis, cause, and response to various treatments. Swartzman and McDermid (1993) studied the impact of contextual cues on the interpretation of and response to physical symptoms. Benson et al (1991) compared Physicians' recognition of and response to child abuse in Northern Ireland and the U.S.A.

[1]Diplomat American Board of Psychiatry and Neurology is a Clinical Assistant Professor of Psychiatry in New Jersey Medical School, University of Medicine and Dentistry of New Jersey at Newark, USA.

[2] Ex professor and chief of the department of Psychiatry of Postgraduate institute of medical Education and Research at Chandigarh, India

*Correspondence : Harish K. Malhotra MD, 33 Overlook Road, Summit, New Jersey 07901, USA. Email nanabhai@pol.net

Wagner et al (2002) studied the degree of ambiguity in the term suicide attempt. They examined 14 expert suicidologists, and 59 general mental health clinicians who either did or did not receive a standard definition of the term. The participants judged whether each of ten vignettes of actual adolescent self-harm behaviors was a suicide attempt. Low levels of agreement were found within each group. Stevens and Brodsky (1995) examined factors hypothesized to influence mental health professionals’ perceptions of dangerousness, predictions of violence, and decisions on patients’ release. The independent variables, manipulated within vignettes, were (a) violence history, (b) paranoid schizophrenia versus nonparanoid schizophrenia, and (c) perceived consequences in terms of liability and publicity.

### Study of treatment practices using vignettes

Farmer and Griffiths (1992) compared factors influencing general practitioners’ and psychiatrists’ decisions regarding patient referral to mental illness services. Allen et al (1988) studied clinical opinion regarding indications for extended psychiatric hospitalization in Menniger clinic and Harding Hospital in the USA.

McDonald-Scott et al (1992) found that the diagnostic communication between doctors and patients differ radically between Japan and Western countries. While over 90% of both groups would inform patients with affective and anxiety disorders of their diagnoses, only 70% of North Americans and less than 30% of Japanese would similarly inform patients with schizophrenia or schizophreniform disorders.

Epstein et al (2001) studied a national sampling of 278 psychiatrists who answered diagnosis and treatment questions for one of four case vignettes with depression and various degrees of medical comorbidity. Tendency to recommend an antidepressant was significantly associated with the psychiatrist being male, being less satisfied with practice, and having a greater percentage of patients on psychotropic medications.

Tiemeier et al (2002) assessed the appropriateness of and variation in intention-to-treat decisions in the management of depression in the Netherlands. They mailed survey with 22 vignettes to a random sample from four professional groups in the Dutch mental healthcare system general practitioners, psychiatrists, psychotherapists, and clinical psychologists. Uhlenhuth et al (1999) focused on the experts’ responses to questions about therapeutic options relevant to seven vignettes describing typical cases of different anxiety disorders. Sices et al (2004) studied factors that influence how primary care physicians’ manage young children with probable developmental delays.

Uncapher and Arenas (2000) mailed one of two case vignettes of a suicidal, depressed patient. The only difference between the two vignettes was the age of the patient (38 or 78 years old) and employment status (employed vs. retired as a factory worker). This study suggests that primary care physicians are capable of recognizing suicidal ideation but are less willing to treat it if the patient is older and retired. Future research needs to determine etiologic factors for this age bias. Saarela and Engestrom (2003) studied differences in management strategies by primary care physicians and psychiatrists in older patients who are depressed. Hirai et al (2003) sent vignettes representing terminally ill cancer patients with anxiety, guilt feelings and dependence related meaninglessness to Japanese health professionals to study their attitudes about the perceived levels of efficacy of different interventions. Green (2003) examined potential differences in the physician’s pain management based on the type of pain, patient demographics and physician characteristics. Tamayo-Sarver et al (2003) developed three clinical vignettes designed to engage physicians’ decision-making processes to prescribe opioid analgesics. The patient’s race/ethnicity was included. Each vignette randomly included or omitted explicit socially desirable information. Weisse et al (2003) examined whether gender or race influences physicians’ pain management decisions. Medical vignettes were used to vary patient gender and race experimentally while holding symptom presentation constant.

Thaver et al (1998) assessed the nature and quality of care provided by 201 practitioners selected from four districts of the slums of Karachi in Pakistan. Vignettes of specific medical problems were used to assess their knowledge and their practice was measured by observing 658 real doctor-patient contacts.

Homs (2003) surveyed the diagnostic procedures and treatment strategies employed in hospitals in the Netherlands for patients with esophageal cancer and the factors affecting the choice of treatments, in particular surgical treatment. Skaner et al (2003) studied heart failure diagnosis in primary health care. It was a clinical judgment analysis study of 40 case vignettes based on authentic patients. Gross et al (2003) investigated why some elderly patients with nonvalvular atrial fibrillation who might benefit from warfarin therapy do not receive it. They identified physicians’ attitudes and beliefs that are associated with their reported use of warfarin in case scenarios. Jorg (2002) sent case vignettes to needs- assessors to investigate agency-related factors predicting allocation of scooters to community –dwelling elderly in the Netherlands. A survey questionnaire containing three medical intensive care clinical vignettes was mailed to critical care physicians by Jain et al (2003). They found significant heterogeneity in selecting an intervention based on pulmonary artery catheter data among intesivists.

### Attitudes of health-care professionals using vignettes

Davidson and Schattner (2003) explored doctors' perceptions of the acceptable limits to self-treatment and identified barriers to doctors seeking appropriate healthcare for themselves.

Chung et al, (2003) investigated how does resident's interactions that occur during rotations influence their learning and practice styles in a residency program. They distributed clinical vignette to all pediatric residents in a teaching hospital, eliciting practice styles for childhood fever and asthma, propensities to order tests for fever, empirically treat fever, diagnose asthma as serious, and aggressively treat asthma.

Hugo (2001) studied mental health professionals' attitudes towards people who have experienced a mental health disorder. Kloss and Lisman (2003) studied attributions of blame perceived by different mental health professionals towards mentally ill and the chemical abuser. Jorm et al, (1997) compared Beliefs of general practitioners, psychiatrists and clinical psychologists about the helpfulness of interventions for mental disorders. Wanless and Jahoda (2002) did a cognitive emotional analysis by studying the responses of staff towards people with mild to moderate intellectual disability and who behave aggressively. The present study involved staff who worked with frequently aggressive clients. They compared different methods of examining the cognitive and emotional responses of staff to aggression; namely, descriptive vignettes and real incidents of aggression which staff could recall.

Lako and Lindenthal (1991) compared the management of confidentiality in general medical practice in the U.S.A. and the Netherlands. Neumann and Olive (2003) studied value systems of general practitioners and psychiatrist by sending them three vignettes describing ethically sensitive scenarios concerning birth control medication for sexually active single women, euthanasia and abortion. Bremberg et al, (2003) assessed how general practitioners would act and how they would justify their choices when facing reluctant and demanding patient. An example is when a pulmonary cancer patient is reluctant to quit smoking. Shah and Mukherjee (2003) surveyed approaches used by psychiatrists to assess capacity to consent for treatment in a London psychiatric trust.

Arnaud et al, (2003) mailed two vignettes to French surgeons. The first one dealt with a man, 46-years-old, with a rectal cancer. The second one dealt with a woman, 50-years-old, with a rectal cancer complicated by a rectovaginal fistula. Questions covered the decision modality and the therapeutic choice for the treatment of locally advanced rectal cancers. Eeles (2003) explored the criteria that nurses use to evaluate spiritual-type experiences reported by patients. Spiritual experiences and psychotic symptoms have many aspects of form and content in common. Despite this, clinicians make judgments about the pathology of these experiences and base care-plans on these judgments. Semi-structured interviews incorporating vignettes of spiritual-type experiences were given to UK mental health nurses.

### Vignettes as teaching and training tool for professional staff

Ruben (2003) constructed vignettes of five children, each with a different form of language disorder as a teaching aid. Butler et al, (1997) saw what effect a half a day postgraduate training course had on the views and knowledge of a group of local GPs on the management of depression in the elderly. The general practitioners attended the course and completed a questionnaire with clinical vignettes before and six weeks after the course. White (2003) studied medical students' learning needs about setting and maintaining social and sexual boundaries. Short answers to a series of vignettes demonstrated conservatism on the part of students when faced with dilemmas. Cacciola et al, (1997) constructed and used case vignettes for Addiction Severity Index (ASI) training. The ASI vignettes, relative to videotaped or live observed interviews, do however provide a brief means of assessing the adequacy of ASI interviewer skills with regard to interviewer severity ratings.

### Attitudes of patient and family towards mental illness using vignettes

Arkar and Eker (1994) studied the influence of having a hospitalized mentally ill member in the family on attitudes toward mental patients in Turkey. Sonuga-Barke and Balding (1993) studied British parents' beliefs about the causes of three forms of childhood psychological disturbance. Swartz et al, (2003) studied attitudes of patients, their families, clinicians and general public about involuntary commitment. They read short vignettes that depicted potential outcomes that were associated alternatively with outpatient commitment and with voluntary treatment.

**Attitudes of general public towards mental illness using vignettes**

Vandello and Cohen (2003) explored how domestic violence may be implicitly or explicitly sanctioned and reinforced in cultures where honor is a salient organizing theme. Study involved participants from Brazil (an honor culture) and the United States responding to written vignettes involving infidelity and violence in response to infidelity.

Marwaha and Livingston (2002) explored and compared the views of White British and Black African-Caribbean older people on depression as an illness, avenues of help and the place of mental health services. Weisman and Lopez (1996) studied family values, religiosity, and emotional reactions to a vignette of schizophrenia in Mexican and Anglo-American cultures.

Hall et al, (1993) in England, Kohn et al, (2000) in Commonwealth of Dominica, Lauber et al, (2003) in Germany, Shulman and Adams (2002) in Britain and Russia, Hugo (2003) in the South Africa, Jorm et al (1997) in Australia, Alem (1999) in Ethiopia, Salomon et al (2004) in six different cultures studied public attitudes towards the mentally ill. Kurihara et al (2000) investigated the differences in public attitudes towards the mentally ill in Bali (Indonesia), a non-industrialized society, and Tokyo (Japan), an industrialized society in Asia. Rost (1993) studied rural-urban differences in stigma and the use of care for vignettes of depressive disorders in USA. Levav et al, (1990) studied mental health attitudes and practices of Soviet immigrants to Israel. Patel et al (1995) in Zimbabwe found that angered ancestral spirits, evil spirits and witchcraft were seen as potent causes of mental illness. Families not only bore the burden of caring for the patient and all financial expenses involved, but were also ostracized and isolated. Arkar and Eker (1992) studied effect of psychiatric labels on attitudes toward mental illness in a Turkish sample. Simonds and Thorpe (2003) studied attitudes toward obsessive-compulsive disorders in undergraduate students. Elliott and Fuqua (2002) studied the acceptability ratings of four interventions targeting trichotillomania (habit reversal, hypnosis, medication, and punishment).

Watson (2004) examined how knowledge that a person has a mental illness influences police officers' perceptions, attitudes, and responses. Police officers viewed persons with schizophrenia as being less responsible for their situation, more worthy of help, and more dangerous than persons for whom no mental illness information was provided.

Frileux et al (2003) studied factors affecting general public's judgment in the acceptability of physician assisted suicide and euthanasia. Kodadek and Feeg (2002) using vignettes, explored how parents approach end-of-life decision making for terminally ill infants. Corrigan et al (2003) surveyed college students to examine the relationships between causal attributions (e.g., controllability, responsibility), familiarity with mental illness, dangerousness, emotional responses (e.g., pity, anger, fear), and helping and rejecting responses.

Alderfer (2001) studied preadolescents who indicated social acceptance of hypothetical children portrayed in vignettes as either chronically ill or healthy and pointed out that social behavior and illness interact to influence the peer acceptance.

Peshkin et al (2003) sent breast cancer specialists vignettes of breast cancer patients to investigate treatment practices in the use of Tamoxifen for chemoprevention. Berlin (2002) studied the impact of diabetes disclosure on perceptions of eating and self-care behaviors. A vignette was developed in which a hypothetical friend engaged in diabetes self-care behaviors during a meal.

**Attitudes of general public towards the treatment of mental illness using vignettes**

Angermeyer and Matschinger (1996) recorded the lay public preferences for various treatments for psychiatric illnesses in Germany.

**Vignettes in the study of patient characteristics**

O'Connor (2003) reported that vignette methodology provided useful method for predicting drop out from an alcohol treatment program. McEvoy et al (1993) gave vignettes to patients with schizophrenia or schizoaffective disorder and to mental health professionals to investigate patients' "insight". Lambert et al (1992) explored Jamaican and American adult perspectives on child psychopathology. The respondents judged vignettes of two children, one with over controlled (e.g., fearfulness) and one with under controlled (e.g., fighting) problems.

**Vignettes in the study of experimental psychology**

Slifer et al (2003) assessed facial emotion encoding and decoding skills in children with and without oral clefts. They were videotaped while listening to a series of brief vignettes designed to evoke facial emotions and while posing prototypic facial expressions. Muris et al (2003) studied anxiety, threat perception abnormalities, and emotional reasoning in nonclinical Dutch children. They used vignettes in which external (i.e., exposure to potential threat cues) and internal (i.e., experience of anxiety responses) information were systematically varied.

Furman and Thompson (2002) examined the influence of teasing on an individual's mood and body satisfaction. They used strategy-written appearance-based scenarios in which another female is the target of teasing comments. Female college students read vignettes that involved a female receiving a comment from another person regarding her appearance or abilities. The findings have implications in etiology of eating disorders.

Richardson et al (2003) developed the KAMA instrument (Knowledge And Management of Abuse), a parallel form vignette-based instrument that tested not only baseline knowledge but also the change in knowledge. Abusive scenarios in vignettes form were adapted from researchers' clinical practice and from the literature. Houben et al (2004) tested the factor structure, reliability and validity of the Health Care Providers' Pain and Impairment Relationship Scale (HC-PAIRS). They sent health care providers vignettes of patients and asking their recommendations for work and physical activity.

Sheridan et al (2003) studied stalking scenarios. The prior relationship and the gender of the stalker and victim were systematically manipulated in order to judge culpability and consequences for the persons involved. Written vignettes were presented to participants who responded. Stalker-victim relationship had three levels: ex-partner, acquaintance and stranger. Thus the effect of the gender of the stalker was investigated.

Cimbora and McIntosh (2003) and Dopke et al, (2003) examined the role of emotion-based moral processes in the committing of delinquent acts by adolescent males with conduct disorder (CD). An Affective Morality Index (AMI) was developed to assess emotional responses to vignettes of delinquent acts. CD groups, as compared to a non-CD group, reported lower levels of guilt and fear and higher levels of excitement and happiness following described transgressions. Grau and Doll (2003) studied the effects of attachment styles on the experience of equity in heterosexual couples' relationships. It involved vignettes describing fictitious characters with typical attachment styles.

### Audio-taped vignettes as a research tool

Galovski et al, (2003) used audio-taped aggressive driving vignettes and non-driving related fearful vignette to investigate the aggressive drivers' systolic blood pressure responsively and the effect of cognitive behavioral treatment. Moore et al (2003) studied the effects of relationship aversive female partner,s behavior on attributions and physiological reactivity of verbally aggressive and non-aggressive males. Participants were presented four audiotape vignettes which depicted hypothetical dating situations in which the female's behavior was relationship aversive or non-relationship aversive. Participants' physiological reactivity (i.e., systolic blood pressure, diastolic blood pressure, and heart rate) was obtained before and after hearing each vignette.

### Vignettes as Trigger films

Hartland et al (2003) used vignettes in audio visual form. Trigger films are 2- to 4-minute vignettes simulating real-life situations that finish abruptly, stimulating participants to analyze situations in a safe environment. They used them as an aid to developing, enabling and assessing anesthesia clinical instructors during their training.

### Research in India using vignettes

The western vignettes were considered inappropriate for research in India and hence fourteen new case histories were developed by Malhotra and Wig (Malhotra, 1973; Malhotra & Wig, 1975). The diagnoses were arrived by the help of Indian psychiatrists, thus called Standard Case Histories. The psychiatrists contacted were the fellows of Indian Psychiatric Society or were the members who had a diploma or a post diploma degree in psychiatry. A total of two hundred three psychiatrists were requested to take part in the study. One hundred and seven psychiatrists agreed to participate. The fourteen case histories along with a questionnaire were sent to them. They completed and returned it. The data were analyzed to assign diagnoses. There were a number of subsequent publications using these Standard Case Histories (nick named "Malhotra-Wig vignettes" for future reference). Sriram and Chandrashekar et al (1990) attempted to develop and standardize case vignettes but only 21 psychiatrists took part in the standardization. Dr. Wig has retired since then and Dr Malhotra has moved to USA. The original text of Malhotra-Wig vignettes is not easily available to future research workers. (Hence a complete text is included in this paper and future researchers will be able to access it easily. )

### The Standard "Malhotra-Wig vignettes"

Malhotra and Wig (1975) reported the detailed diagnostic breakdown of the fourteen Standard Malhotra-Wig vignettes including the percentage of psychiatrist supporting the majority diagnoses along with statistical analysis. One vignette of a normal person was included for control if needed. The data has been summarized in table 1.

Table 1

**Percentage distribution of Indian psychiatrists by diagnostic agreement about the fourteen Standard Case Histories, "Malhotra-Wig Vignettes".**

| Standard Case History No. | Total diagnoses offered | Diagnosis with maximum support | Percentage of Psychiatrists Supporting the diagnosis |
|---|---|---|---|
| 1 | 3 | Schizophrenia | 98.2 |
| 2 | 8 | Schizophrenia, Paranoid type | 79.4 |
| 3 | 6 | Hysterical neurosis | 94.4 |
| 4 | 7 | Manic depressive Psychosis, depressive | 65.4 |
| 5 | 6 | Behavior disorder | 51.4 |
| 6 | 8 | Normal, No psychiatric diagnosis | 30.4 |
| 7 | 5 | Physiological night emission | 84.1 |
| 8 | 4 | Obsessive compulsive neurosis | 97.3 |
| 9 | 5 | Anxiety neurosis | 897 |
| 10 | 10 | Psychogenic impotence | 70.0 |
| 11 | 4 | Manic depressive psychosis, mania | 93.4 |
| 12 | 4 | Alcoholic addiction | 92,2 |
| 13 | 4 | Dementia | 89:3 |
| 14 | 7 | Antisocial personality disorder | 84.6 |

Standard Case Histories for attitudinal research in India. Adopted from Malhotra and Wig (1975).

***Review of the research using vignettes in India***

**Classification of Psychiatric Disorders in India**

Malhotra et al (1974) reported the wide variety of terminology used to describe psychiatric syndromes. They sent the fourteen case histories to psychiatrists and asked them to diagnose them. They pointed that multiple terms were used to describe a diagnosis. The terminology significantly deviated from International Classification of Diseases. They pointed to the need for improvement in the way Indian psychiatrists used diagnostic terminology.

**Sexual disorders in India**

Malhotra and Wig (1975) studied attitudes of general population about loss of semen and applied those findings in explaining the causation of Dhat syndrome. They read out Standard Malhotra-Wig vignette number seven, a case of a nocturnal emission in a teenager. They interviewed members of general public and reported that the attitudes about harmful effects of semen loss are prevalent across all socioeconomic classes. Such attitudes were more prevalent in lower socio economic classes. Malhotra et al (1981) sent Standard Malhotra-Wig vignette number seven, the teenager, to "sex specialists" collected from advertisements from newspapers. They brought to light the modus operandi of these quacks. Instead of helping to calm the anxiety about a normal function of body, they fan the fears by giving misleading information and exploit the ignorant.

**Attitudes of general population in India**

Malhotra and Wig (1975) studied attitudes of the general population about using a general physician when they encounter a deviant behavior. They pointed out that majority of Indians looked towards a general practitioner for most of their problems. The knowledge of the use of psychiatrists was less frequent. Malhotra et al (1976) studied public attitudes about, how does the public manage deviant behavior and how does socioeconomic class effect this perception. Malhotra et al. (1977) read out the Standard Malhotra-Wig vignette of a child and studied public opinion about childhood behavior disturbance and the child guidance clinic in India. Malhotra & Wig (1977) studied the attitudes of psychiatrists as to where do they want to treat psychiatric patients. They studied the attitudes toward the use of outpatient, general hospital psychiatric unit and state hospital. Murthy (1977) studied rural community attitudes towards mental disorders using his vignettes. Segal (1992) revealed that there was negligible variation in perceptions of the severity of different forms of abuse among Indian mental health workers. Cross-cultural comparisons with a U.S. study indicated some differences in perceptions. The Indian view holds the child as parental property, subject to discipline as parents find appropriate. Battering of children in India is not seen as detrimental to the child.

### Epidemiology in India

Harding et al, (1983) and Timothy et al, (1983) describe the development and use of vignettes for the WHO Collaborative Study on Strategies for Extending Mental Health Care in the Development of new research methods. These vignettes were based on Malhotra and Wig (1975). Wig et al, (1980) used the vignettes, based on the Standard Malhotra-Wig vignettes, in finding community reactions to mental disorders. They read out their vignettes to a key informant in a village. The study was conducted in three developing countries. Murthy et al, (1978) similarly used vignettes in case identification .

### Clinical psychology in India

Malhotra et al, (1975) used these case hisotries as a projective technique in a psychological test. Indian masses are more used to spoken words. Hence instead of showing them pictures, they read out the Standard Case Hisotries and asked questions to explore psychological conflicts.

### Teaching and Training in India

Later, other authors constructed their own case hisotries. Shamsunder et al, (1985). studied training of general practitioners in psychiatry . Manickam (1990) studied empathy in professionals and trained lay counsellors using hypothetical situations.

Murthy and Arora (1976) studied attitude change in medical postgraduates following short-term training in psychiatry. Sriram and Moily et al (1990) compared errors in clinical judgment before and after training of primary health care medical officers in mental health care.

### Summary:

The international literature has been reviewed with a bird's eye view of the variety of ways the vignettes have been used in different fields of medicine. Indian psychiatric literature has been reviewed to focus on vignette research in India. The full text of "Malhotra- Wig vignettes" in English and Hindi has been provided for use by future research workers. Dr. Harish K Malhotra and Professor N.N. Wig hereby give permission to any future research worker to use the vignettes without the need for prior permission from the authors.

### REFERENCES

Alderfer MA, Wiebe DJ, Hartmann DP., (2001) Social behavior and illness information interact to influence the peer acceptance of children with chronic illness. Br J Health Psychol. 6(Pt 3):243-55.

Alem A, Jacobsson L, Araya M, Kebede D, Kullgren G., (1999) How are mental disorders seen and where is help sought in a rural Ethiopian community? A key informant study in Butajira, Ethiopia. Acta Psychiatr Scand Suppl. ;397:40-7.

Allen JG, Scovern AW, Logue AM, Coyne L., (1988) Indications for extended psychiatric hospitalization: a study of clinical opinion. Compr Psychiatry. 29(6):604-12.

Angermeyer MC, Matschinger H., (1996) Public attitude towards psychiatric treatment. Acta Psychiatr Scand. 94(5):326-36.

Arkar H, Eker D. (1994) Effect of psychiatric labels on attitudes toward mental illness in a Turkish sample. Int J S of Psychiatry. Autumn; 40(3): 205-13.

Arkar H, Eker D., (1992) Influence of having a hospitalized mentally ill member in the family on attitudes toward mental patients in Turkey. Soc Psychiatry Psychiatr Epidemiol. 27(3):151-5.

Arnaud S, Houvenaeghel G, Julian-Reynier C, Moutardier V, Delpero JR, Moatti JP., (2003) [Advanced rectal cancer: attitudes of French surgeons][Article in French] Ann Chir. 128(5):293-300; discussion 301-2.

Bates LW, Lyons JA, Shaw JB., (2002) Effects of brief training on application of the Global Assessment of Functioning Scale. Psychol Rep. 91(3 Pt 1):999-1006.

Benson DE, Swann A, O'Toole R, Turbett JP., (1991) Physicians' recognition of and response to child abuse: Northern Ireland and the U.S.A. Child Abuse Negl. 15(1-2):57-67.

Berlin KS, Sass DA, Davies WH, Hains AA., (2002) Impact of diabetes disclosure on perceptions of eating and self-care behaviors. Diabetes Educ. 28(5):809-16.

Bremberg S, Nilstun T, Kovac V, Zwitter M., (2003) GPs facing reluctant and demanding patients: analysing ethical justifications. Fam Pract. 20(3):254-61.

Butler R, Collins E, Katona C, Orrell M., (1997) Does a teaching programme improve general practitioners' management of depression in the elderly? J Affect Disord. 46(3):303-8.

Cacciola JS, Alterman AI, Fureman I, Parikh GA, Rutherford MJ., (1997) The use of case vignettes for Addiction Severity Index training. J Subst Abuse Treat. 14(5):439-43.

Chung PJ, Chung J, Shah MN, Meltzer DO., (2003) How do residents learn? The development of practice styles in a residency program. Ambul Pediatr. 3(4):166-72.

Cimbora DM, McIntosh DN., (2003) Emotional responses to antisocial acts in adolescent males with conduct disorder: a link to affective morality. J Clin Child Adolesc Psychol. 32(2):296-301.

Corrigan P, Markowitz FE, Watson A, Rowan D, Kubiak MA., (2003) An attribution model of public discrimination towards persons with mental illness. J Health Soc Behav. 44(2):162-79.

Davidson SK, Schattner PL., (2003) Doctors' health-seeking behavior: a questionnaire survey. Med J Aust. 179(6):302-5.

Dopke CA, Lundahl BW, Dunsterville E, Lovejoy MC., (2003) Interpretations of child compliance in individuals at high- and low-risk for child physical abuse. Child Abuse Negl. 27(3):285-302.

Eeles J, Lowe T, Wellman N., (2003) Spirituality or psychosis?—An exploration of the criteria that nurses use to evaluate spiritual-type experiences reported by patients. Int J Nurs Stud. 40(2):197-206.

Elliott AJ, (2002) Fuqua WR Acceptability of treatments for trichotillomania. Effects of age and Severity Behav Modif. 26(3):378-99.

Epstein SA, Gonzales JJ, Weinfurt K, Boekeloo B, Yuan N, Chase G., (2001) Are psychiatrists' characteristics related to how they care for depression in the medically ill? Results from a national case-vignette survey Psychosomatics. 42(6):482-9

Farmer AE, Griffiths H., (1992) Labeling and illness in primary care: comparing factors influencing general practitioners' and psychiatrists' decisions regarding patient referral to mental illness services. Psychol Med. 22(3):717-23.

Flanagan EH, Blashfield RK., (2003) Gender bias in the diagnosis of personality disorders: the roles of base rates and social stereotypes. J Personal Disord. 17(5):431-46.

Frileux S, Lelievre C, Munoz Sastre MT, Mullet E, Sorum PC., (2003) When is physician assisted suicide or euthanasia acceptable? J Med Ethics. 29(6):330-6

Furman K, Thompson JK., (2002) Body image, teasing, and mood alterations: an experimental study of exposure to negative verbal commentary. Int J Eat Disord. 32(4):449-57.

Galante AC, Aranha JA, Beraldo L, Pel NT., (2003) [The vignette as a strategy for data collection in nursing research][Article in Portuguese] Rev Lat Am Enfermagem. 11(3):357-63. Epub 2003, 02.

Galovski TE, Blanchard EB, Malta LS, Freidenberg BM., (2003) The psychophysiology of aggressive drivers: comparison to non-aggressive drivers and pre- to post-treatment change following a cognitive-behavioral treatment. Behav Res Ther. 41(9):1055-67.

Gould D. Using, (1996) vignettes to collect data for nursing research studies: how valid are the findings? Clin Nurs., 5(4):207-12

Grau I, Doll J., (2003) Effects of attachment styles on the experience of equity in heterosexual couples relationships. Exp Psychol. 50(4):298-310.

Green CR, Wheeler JR, LaPorte F., (2003) Clinical decision making in pain management: Contributions of physician and patient characteristics to variations in practice. J Pain. 4(1):29-39.

Gross CP, Vogel EW, Dhond AJ, Marple CB, Edwards RA, Hauch O, Demers EA, Ezekowitz M., (2003) Factors influencing physicians' reported use of anticoagulation therapy in nonvalvular atrial fibrillation: a cross-sectional survey. Clin Ther. 25(6):1750-64.

Hall P, Brockington IF, Levings J, Murphy C., (1993) A comparison of responses to the mentally ill in two communities. Br J Psychiatry. 162:99-108.

Harding TW, Climent CE, Diop M, Giel R, Ibrahim HH, Murthy RS, Suleiman MA, and Wig NN, (1983) The WHO collaborative study on strategies for extending mental health care, II: The development of new research methods. Am J Psychiatry 140: 1474-1480.

Hartland W, Biddle C, Fallacaro M., (2003) Accessing the living laboratory: trigger films as an aid to developing, enabling, and assessing anesthesia clinical instructors. AANA J. 71(4):287-91.

Hasui C, Sugiura T, Tanaka E, Sakamoto S, Sugawara M, Kitamura T, Aoki Y., (1999) Reliability of childhood mental disorder: diagnoses by Japanese psychologists. Psychiatry Clin Neurosci. 53(1):57-61.

Hirai K, Morita T, Kashiwagi T., (2003) Professionally perceived effectiveness of psychosocial interventions for existential suffering of terminally ill cancer patients. Palliat Med. 17(8):688-94.

Hjortso S, Butler B, Clemmesen L, Jepsen PW, Kastrup M, Vilmar T, Bech P., (1989). The use of case vignettes in studies of interrater reliability of psychiatric target syndromes and diagnoses. A comparison of ICD-8, ICD-10 and DSM-III. Acta Psychiatr Scand. 80(6):632-8

Homs MY, Steyerberg EW, Tilanus HW, van der Gaast A, Kuipers EJ, Siersema PD., (2003) Diagnosis and treatment of esophageal cancer in the Netherlands: wide variation in the second line [Article in Dutch] Ned Tijdschr Geneeskd. 25; 147(43):2128-33.

Houben RM, Vlaeyen JW, Peters M, Ostelo RW, Wolters PM, Stomp-van den Berg SG., (2004) Health care providers' attitudes and beliefs towards common low back pain: factor structure and psychometric properties of the HC-PAIRS. Clin J Pain. 20(1):37-44.

Hsieh DK, Kirk SA., 2003 The effect of social context on psychiatrists' judgments of adolescent antisocial behavior. J Child Psychol Psychiatry., 44(6):877-87.

Hughes R, Huby M., (2002) The application of vignettes in social and nursing research. J Adv Nurs., 37(4):382-6

Hugo CJ, Boshoff DE, Traut A, Zungu-Dirwayi N, Stein DJ., (2003) Community attitudes toward and knowledge of mental illness in South Africa. Soc Psychiatry Psychiatr Epidemiol. 38(12):715-9.

Hugo M., (2001) Mental health professionals' attitudes towards people who have experienced a mental health disorder. J Psychiatr Ment Health Nurs. 8(5):419-25.

Jain M, Canham M, Upadhyay D, Corbridge T., (2003) Variability in interventions with pulmonary artery catheter data. Intensive Care Med. 29(11):2059-62.

Jorg F, Borgers N, Schrijvers AJ., 2003 Client needs assessor and agency-related factors predicting allocation of electric scooters to community-dwelling elderly in The Netherlands: a Vignette study. Disabil Rehabil. 22; 25(14):761-70.

Jorm AF, Korten AE, Jacomb PA, Rodgers B, Pollitt P., (1997) Beliefs about the helpfulness of interventions for mental disorders: a comparison of general practitioners, psychiatrists and clinical psychologists. Aust N Z J Psychiatry. 31(6):844-51.

Jorm AF, Korten AE, Rodgers B, Pollitt P, Jacomb PA, Christensen H, Jiao Z., (1997) Belief systems of the general public concerning the appropriate treatments for mental disorders. Soc Psychiatry Psychiatr Epidemiol. 32(8):468-73.

Kee F, Jenkins J, McIlwaine S, Patterson C, Harper S, Shields M., (2003) Fast and frugal models of clinical judgment in novice and expert physicians. Med Decis Making. 23(4):293-300.

Kitamura T, Shima S, Sakio E, Kato M., (1989) Psychiatric diagnosis in Japan. 2. Reliability of conventional diagnosis and discrepancies with Research Diagnostic Criteria diagnosis. Psychopathology. 22(5):250-9.

Kloss JD, Lisman SA., (2003) Clinician attributions and disease model perspectives of mentally ill chemically addicted patients: a preliminary investigation. Subst Use Misuse. 38(14):2097-107.

Kodadek MP, Feeg VD., (2002) Using vignettes to explore how parents approach end-of-life decision making for terminally ill infants. Pediatr Nurs. 28(4):333-40, 343.

Kohn R, Sharma D, Camilleri CP, Levav I., (2000) Attitudes towards mental illness in the Commonwealth of Dominica. Rev Panam Salud Publica. 7(3):148-54.

Kurihara T, Kato M, Sakamoto S, Reverger R, Kitamura T., (2000) Public attitudes towards the mentally ill: a cross-cultural study between Bali and Tokyo. Psychiatry Clin Neurosci. 54(5):547-52.

Lako CJ, Lindenthal JJ., (1991) The management of confidentiality in general medical practice: a comparative study in the U.S.A. and The Netherlands. Soc Sci Med. 32(2):153-7.

Lambert MC, Weisz JR, Knight F, Desrosiers MF, Overly K, Thesiger C., (1992) Jamaican and American adult perspectives on child psychopathology: further exploration of the threshold model. J Consult Clin Psychol. 60(1):146-9.

Lauber C, Nordt C, Falcato L, Rossler W., (2003) Do people recognize mental illness? Factors influencing mental health literacy. Eur Arch Psychiatry Clin Neurosci. 253(5):248-51.

Levav I, Kohn R, Flaherty JA, Lerner Y, Aisenberg E., (1990) Mental health attitudes and practices of Soviet immigrants. Isr J Psychiatry Relat Sci. 27(3):131-44.

Malhotra H. K., Inam, AS, Wig N. N. (1977). Public opinion and the child guidance clinic in India. Indian Journal of Psychiatry, 19, 14-19.

Malhotra H. K., Wig N. N. (1975) Vignettes for attitudinal research in Psychiatry. Indian Journal of Psychiatry, 17, 195-199.

Malhotra H. K., Wig N. N. (1975). The General physician and the psychiatric patient. Indian Journal of Psychiatry, 17, 191-194.

Malhotra H. K., Wig N. N., Inam, S. A. (1976). How does the public manage deviant behavior? Indian Journal of Psychiatry, 18, 95-101.

Malhotra H. K., Wig N. N., Pershad D. (1974). Diagnostic Synonyms in Psychiatry. Journal of Clinical Psychiatry, 2, 83-86.

Malhotra H. K., Wig N. N., Verma SK. (1975) A simple projective technique for use in India: Initial experiments. Indian Journal of Clinical Psychology, 2,123-127.

Malhotra, H. K. (1973) “A Study of the concept of Mental Illness in the General Population. “A thesis submitted to Post Graduate Institute of Medical Education and Research, Sector 12, Chandigarh, India for the degree of M D in Psychiatry.

Malhotra, H. K. and Wig, N. N., (1975) Dhat Syndrome: A Culture-Bound Sex Neurosis of the Orient. Archives of Sexual Behavior, 4, 5, 519-528.

Malhotra, H. K., Inam, S. A. and Wig, N. N. (1981) The Self-Advertising Sex Specialist: Professional or Quack? Indian Journal of Clinical Psychology, 8, 1-7.

Malhotra, H. K., Wig, N. N. (1977) Where do we want to treat our psychiatric patients? Indian Journal of Psychiatry, 19, 20-26.

Manickam, L. S. S. (1990) Empathy: A comparative study of professionals and trained lay counselors using hypothetical situations. Indian Journal of Psychiatry. 32, 1, 83-88

Marwaha S, Livingston G., (2002) Stigma, racism or choice. Why do depressed ethnic elders avoid psychiatrists? Affect Disord. 72(3):257-65.

McDonald-Scott P, Machizawa S, Satoh H., (1992) Diagnostic disclosure: a tale in two cultures. Psychol Med. 22(1):147-57.

McEvoy JP, Schooler NR, Friedman E, Steingard S, Allen M. (1993). Use of psychopathology vignettes by patients with schizophrenia or schizoaffective disorder and by mental health professionals to judge patients’ insight. Am J Psychiatry; 150(11):1649-53.

Moore TM, Stuart GL, Eisler RM, Franchina JJ., (2003) The effects of relationship aversive female partner behavior on attributions and physiological reactivity of verbally aggressive and non-aggressive males. Violence Vict. 18(1):95-106.

Muris P, Merckelbach H, Schepers S, Meesters C., (2003) Anxiety, threat perception abnormalities, and emotional reasoning in nonclinical Dutch children. J Clin Child Adolesc Psychol. 32(3):453-9.

Murthy RS, Kala R., Wig N. N. (1978) Mentally ill in a rural community: Some initial experiences in case identification and management Indian Journal of Psychiatry, 20, 2, 143-147.

Murthy, R. S., Arora, M. (1976) Attitude change in medical postgraduates following short-term training in psychiatry. Indian Journal of Clinical Psychology, 3, 165-169.

Murthy, R.S. (1977) Rural Community attitudes to mental disorders: Preliminary findings Malhotra H.. Indian Journal of Clinical Psychology, 4,141-144.

Neumann JK, Olive KE., (2003) Absolute versus relative values: effects on family practitioners and psychiatrists. South Med J. 96(5):452-7.

O’Connor SM, Davies JB, Heffernan DD, van Eijk R., (2003) An alternative method for predicting attrition from an alcohol treatment programme. Alcohol Alcohol. 38(6):568-73

Oliver P, Cooray S, Tyrer P, Cicchetti D., (2003) Use of the Global Assessment of Function scale in learning disability. Br J Psychiatry Suppl. 44:S32-5

Patel V, Musara T, Butau T, Maramba P, Fuyane S., (1995) Concepts of mental illness and medical pluralism in Harare. Psychol Med. 25(3):485-93.

Peabody JW, Luck J, Glassman P, Dresselhaus TR, Lee M., (2000) Comparison of vignettes, standardized patients, and chart abstraction: a prospective validation study of 3 methods for measuring quality. AMA., 5; 283(13):1715-22

Peshkin BN, Isaacs C, Finch C, Kent S, Schwartz MD., (2003) Tamoxifen as chemoprevention in BRCA1 and BRCA2 mutation carriers with breast cancer: a pilot survey by physicians. J Clin Oncol. 1; 21(23): 4322-8.

Richardson B, Kitchen G, Livingston G., (2003) Developing the KAMA instrument (knowledge and management of abuse). Age Ageing. 32(3): 286-91.

Rost K, Smith GR, Taylor JL (1993) Rural-urban differences in stigma and the use of care for depressive disorders. J Rural Health. 9(1):57-62.

Ruben RJ., (2003) Five children—vignettes of language disorders. Int J Pediatr Otorhinolaryngol. 67 Suppl 1:S125-30.

Saarela T, Engestrom R., (2003) Reported differences in management strategies by primary care physicians and psychiatrists in older patients who are depressed. Int J Geriatr Psychiatry. 18(2):161-8.

Salomon JA, Tandon A, Murray CJ., (2004) Comparability of self rated health: cross sectional multi-country survey using anchoring vignettes. BMJ. 31; 328(7434):258

Segal UA., (1992) Child abuse in India: an empirical report on perceptions. Child Abuse Negl. 16(6):887-908.

Shah A, Mukherjee S., (2003) Ascertaining capacity to consent: a survey of approaches used by psychiatrists. Med Sci Law. 43(3):231-5.

Shamasundar C, John J, Reddy PR, Kaliaperumal V.G., Verghese A, Chandramauli, Isaac M.K. (1989). Clinical vignettes for assessment of training general practitioners in psychiatry. Indian Journal of Psychiatry, 31(4), 280-284.

Sheridan L, Gillett R, Davies GM, Blaauw E, Patel D., (2003) ‘There’s no smoke without fire’: are male ex-partners perceived as more ‘entitled’ to stalk than acquaintance or stranger stalkers? Br J Psychol. 94(Pt 1):87-98.

Shulman N, Adams B., (2002) A comparison of Russian and British attitudes towards mental health problems in the community. Int J Soc Psychiatry. 48(4):266-78.

Sices L, Feudtner C, McLaughlin J, Drotar D, Williams M., (2004) How do primary care physicians manage children with possible developmental delays? A national survey with an experimental design. Pediatrics. 113(2):274-82.

Simonds LM, Thorpe SJ., (2003) Attitudes toward obsessive-compulsive disorders—an experimental Investigation. Soc Psychiatry Psychiatr Epidemiol. 38(6):331-6.

Skaner Y, Bring J, Ullman B, Strender LE., (2003) Heart failure diagnosis in primary health care: clinical characteristics of problematic patients. A clinical judgement analysis study. BMC Fam Pract. 4(1):12.

Slifer KJ, Diver T, Amari A, Cohn JF, Hilley L, Beck M, McDonnell S, Kane A., (2003) Assessment of facial emotion encoding and decoding skills in children with and without oral clefts. J Craniomaxillofac Surg. 31(5):304-15.

Sonuga-Barke EJ, Balding J., (1993) British parents’ beliefs about the causes of three forms of childhood psychological disturbance. J Abnorm Child Psychol. 21(4):367-76.

Sriram TG, Chandrashekar CR, Isaac MK, SrinivasaMurthy R, Kishore Kumar KV, Moily S, Shanmugham V., (1990) Development of case vignettes to assess the mental health training of primary care medical officers. Acta Psychiatr Scand. 82(2):174-7.

Sriram TG, Moily S, Kumar GS, Chandrashekar CR, Isaac MK, Murthy RS. (1990) Training of primary health care medical officers in mental health care. Errors in clinical judgment before and after training. Gen Hosp Psychiatry. Nov; 12(6):384-9.

Stelmachers ZT, Sherman RE. (1990) Use of case vignettes in suicide risk assessment. Suicide Life Threat Behav. Spring; 20(1):65-84

Stevens HB, Brodsky SL., (1995) Perceived consequences to the predictor: a variable in the release of psychiatric patients. 76(3 Pt 2):1371-8

Swartz MS, Swanson JW, Wagner HR, Hannon MJ, Burns BJ, Shumway M., (2003) Assessment of four stakeholder groups’ preferences concerning outpatient commitment for persons with schizophrenia. Am J Psychiatry. 160(6):1139-46.

Swartzman LC, McDermid AJ., (1993) The impact of contextual cues on the interpretation of and response to physical symptoms: a vignette approach. J Behav Med. 16(2):183-98.

Tamayo-Sarver JH, Dawson NV, Hinze SW, Cydulka RK, Wigton RS, Albert JM, Ibrahim SA, Baker DW., (2003) The effect of race/ethnicity and desirable social characteristics on physicians’ decisions to prescribe opioid analgesics. Acad Emerg Med. 10(11):1239-48.

Thaver IH, Harpham T, McPake B, Garner P., (1998) Private practitioners in the slums of Karachi: what quality of care do they offer? Soc Sci Med. 46(11):1441-9.

Tiemeier H, De Vries WJ, Van Het Loo M, Kahan JP, Klazinga N, Grol R, Rigter H., (2002) Guideline adherence rates and interprofessional variation in a vignette study of depression. Qual Saf Health Care. 11(3):214-218

Uhlenhuth EH, Balter MB, Ban TA, Yang K. (1999) International study of expert judgment on therapeutic use of benzodiazepines and other psychotherapeutic medications: VI. Trends in recommendations for the pharmacotherapy of anxiety disorders, 1992-1997 Depress Anxiety. 9(3):107-16

Uncapher H, Arean PA., (2000) Physicians are less willing to treat suicidal ideation in older patients. J Am Geriatr Soc. 48(2):188-92.

Vandello JA, Cohen D., (2003) Male honor and female fidelity: implicit cultural scripts that perpetuate domestic violence. J Pers Soc Psychol. 84(5):997-1010.

Wagner BM, Wong SA, Jobes DA., (2002) Mental health professionals’ determinations of adolescent suicide attempts. Suicide Life Threat Behav. Fall; 32(3):284-300

Wanless LK, Jahoda A., (2002) Responses of staff towards people with mild to moderate intellectual disability who behave aggressively: a cognitive emotional analysis. J Intellect Disabil Res. 46(Pt 6):507-16.

Watson AC, Corrigan PW, Ottati V., (2004) Police officers’ attitudes toward and decisions about persons with mental illness. Psychiatr Serv. 55(1):49-53.

Weisman AG, Lopez, (1996) SR Family values, religiosity, and emotional reactions to schizophrenia in Mexican and Anglo-American cultures Fam Process. 35(2):227-37.

Weisse CS, Sorum PC, Dominguez RE., (2003) The influence of gender and race on physicians’ pain management decisions. J Pain. 4(9):505-10.

White GE., (2003) Medical students’ learning needs about setting and maintaining social and sexual boundaries: a report. Med Educ. 37(11): 1017-9.

Wig, N.N., Suleiman, M.A., Routledge, R, et al (1980) community reactions to mental disorders; a key informant study in three developing countries. Acta Psychiatrica Scandinavica, 61:111-126.

### APPENDIX

### English Version of “Malhotra-Wig vignettes”

### Malhotra-Wig vignette No.1

Bimla is a young girl of twenty. Three years ago she became very quiet. She spends most of her time sitting alone. She does not talk to anybody. She does not answer her parents if they talk to her. She has stopped taking care of her personal hygiene. She does not clean up after herself or do any housework. Sometimes she is seen laughing or weeping to herself. She occasionally appears to be making faces as though she was conversing with someone. She is sometimes found naked. She is afraid of other people.

### Malhotra-Wig vignette No 2

Ram Parkash is 30 years old. He works as an assistant in a shop in the grain market. During the past few months he has become quarrelsome. He accuses his friends of poisoning his food. He abuses them with vulgar language. He is preoccupied with the idea that his employer suspects him of being dishonest. He hears imaginary whistles, which he attributes to the police. He suspects people surrounding him of being plain-clothes police, who are keeping a watch over him. Many people have tried to convince him that this is all in his imagination, but he remains unconvinced. He sleeps poorly. He has started neglecting his appearance. His friends feel that he is a changed person. When others are talking, he believes they are talking about him.

### Malhotra-Wig vignette No 3

Tara is a twenty year-old married woman. She has been married for three years. Her husband is in the army; hence, most of the time he is away. Tara’s mother-in-law is quite irritable and quarrelsome. She taunts Tara, saying that Tara is infertile. Tara feels very badly about it. Tara is an illiterate girl.

Tara is a worshipper of Goddess Durga. Whenever there is a Kirtan in the neighborhood, Tara must attend it. During the Kirtan, Tara will close her eyes and become unaware of others. She starts breathing rapidly. Her face assumes a peculiar expression. She makes strange shrieking sounds and moves her head violently. Sometimes she strikes the ground with her hands and bangs her head on the ground. This lasts for 30-40 minutes. She is all right afterwards except for a slight weakness that lasts for a few hours. She goes home. She takes a glass of hot milk and becomes busy in her usual routine.

### Malhotra-Wig vignette No. 4

Gurmeet Singh is a farmer aged 40. He is a married man with three school-aged children. Gurmeet has always been a social, sweet tempered and happy-go-lucky man. He is a loving father to his children.

For several weeks, Gurmeet has been very sad. He worries over minor details. He does not sleep at night. He blames himself for every small mistake. Every joke or casual remark cuts him to the quick. He has suicidal ideas. Once he was saved from drowning in the village well after he jumped in it to die.

**Malhotra-Wig vignette No. 5**

Ashok is a five-year-old boy. He is the first child of his parents and has been brought up with great affection and care. Six months ago, after due religious ceremonies, he was admitted in school. It was a happy occasion and his parents distributed sweets in the neighborhood. Ashok was enjoying school.

Three months ago, a younger brother was born. Ashok became irritable and weepy after the birth of his younger brother. He is giving his parents a lot of difficulties in going to school. He says that he cannot go because he has pain in his abdomen. He has become stubborn and demanding. Sometimes he slips away from school and comes home. At night, he insists on sleeping with his mother. He is afraid to be alone. At times, his parents have found it necessary to spank him for his bad behavior.

**Malhotra-Wig vignette No. 6**

Somenath is a farmer. He owns about 10 acres of land. He has ten buffaloes. His wife is a good-natured lady. Somenath and she both are very fond of each other. Both love their children. Somenath toils hard in the fields every day. In the evening he listens to Radio music. He went out with his friends a few months back. His friends insisted on his gambling also, which he did and lost Rs.100/-. After that, he has pledged never to drink again. For the last few days, he has been worried about the failure of the rain. He has sown his seed. He has been thinking that if it does not rain in time, there will be no crop this year and there will be great difficulty.

**Malhotra-Wig vignette No. 7**

Narinder is a student of the 10$^{th}$ class. He is quite a good student. He is respectful of his elders and loving to those younger. He likes playing football. He is considered one the of the better football players at his school. His mother noticed semen marks on his pajamas when she was doing the laundry. She feels that Narinder's clothes were soiled while he was sleeping. No change has been noticed in Narinder's behavior. He has never complained of any problem to anyone about his health.

**Malhotra-Wig vignette No. 8**

Raj Dulari is Ramlal's wife. For the last three years she has become very peculiar about cleanliness. She washes her hands repeatedly up to 15 times after going to the toilet. She even rubs the tap with ash to clean it. She is preoccupied with the idea of her hands being dirty. She has become very slow in her daily tasks. She takes a long time preparing breakfast for her husband with the result that her husband Ramlal is late for work. She knows that she has lost control of her actions; she realizes the stupidity of her actions; she feels very badly about it.

**Malhotra-Wig vignette No. 9**

Kishan Singh is a young man of nineteen. He had been doing very well in his studies. His examinations are approaching. He has started feeling nervous. He says that he cannot understand what he reads. In spite of his best efforts, he cannot retain anything. He gets severe palpitations. His hands perspire. He cannot write well because of trembling of his hands. He sleeps poorly.

**Malhotra-Wig vignette No. 10**

Piyare Lal is a simple and religious man. He is not very social. He is busy with his work. He does not accept any bribes. He goes to temple every Tuesday to give offerings to God. His wife is even more religious than Piyare. She is a dominating figure in the family. They have been married for the last five years. They have one three-year-old son. A few days ago his wife went to visit her parents. Piyare Lal was left alone. He felt bored in the lonely house. For a change he went to see a movie. He returned late at night. While serving him dinner, the young female cook gave him hints. Piyare lost himself and slept with the cook that night. In the morning he repented badly. After some days his wife returned and Piyare attempted sexual relations with his wife. He could not achieve an erection.

**Malhotra-Wig vignette No. 11**

Pritam Singh has been working as a chowkidar in a village for the last ten years. He has been a good worker. He used to talk to everybody with respect and courtesy. For the last 10 days it has been noticed that he is unusually happy. He used to be a soft spoken and obedient person, but now he talks boisterously and laughs heartily. People wonder whether he is the same person they used to know. He has been telling everybody that his services are needed urgently in the armed forces and that he will be leaving his present job. He discusses his plans to build a big palace in the village. He has spent most of his savings in offering drinks to his friends. He does not sleep well. He appears full of youth, life and vigor. Once he starts, he does not stop talking. He has stopped going to his duties because he feels that his present job is below his dignity. His wife has remarked that Pritam Singh is a completely changed person and he has forgotten that he is the humble village chowkidar. He now talks of things which are obviously beyond his reach.

**Malhotra-Wig vignette No. 12**

Bant Ram has been drinking for the last eight years. To start with, he drank only in company or when his friends from outside the village visited him. For the last two years, he has been drinking regularly. On the whole he takes two bottles of country liquor a day. He starts drinking early in the morning. His last drink is just before he goes to sleep. He gets irritated and starts fighting if anyone points out to him that he should not drink so much. The drinks have cost him heavily in terms of economy. He had to sell even ornaments of his wife to satisfy his craving for liquor. On account of this, there are frequent fights between him and his wife. If some day he has to go without alcohol, he feels utterly exhausted, cannot work and gets trembling in his hands.

**Malhotra-Wig vignette No. 13**

Mohan Lal is now over seventy five years of age. He has lost all his teeth and his vision is impaired. He had been a well-behaved gentleman and was always neatly dressed. However, a marked change has been noticed in his behavior over the years. He does not care about the cleanliness of his clothes and appearance. He forgets easily and misplaces things. When he cannot find something, he becomes very angry and abusive. He accuses those around him of having stolen his property. There are times when he weeps bitterly, like a child. Many times he has forgotten his way back home and is found wandering in the street. He talks more than he should. Sometimes he cracks vulgar jokes. Sometimes he passes bowel movements in his room.

**Malhotra-Wig vignette No. 14**

Santosh Kumar had been wetting his bed until thirteen years of age. He had lost his father when he was just two years old. His mother had to remain out of the home for most of the time. She had to earn some money to make ends meet. Consequently, she could not devote enough time to Santosh Kumar. He grew up to become a source of trouble for his mother. It was always a problem to send him off to school. Often he would play truant from the school and spend his time watching cinema posters or following the snake charmer. When he was in his $4^{th}$ standard he started picking cigarette butts from the road and smoking them in the company of his friends. His mother tried her best to get him educated but Santosh firmly refused to go to school in eighth class.

Now he is twenty-three. He has been working in a shop. Generally he is late in going to work and sometimes he does not go to work for a day or two. Once his employer gave him Rs.200/- for a bank deposit. Santosh disappeared with the money and returned after five days without the money. Later it became known that he had gone to another city and stayed in a hotel. He had taken a boy of twelve with him and had indulged in sodomy.
